# Native and Non-Native *Bemisia tabaci* NAFME Haplotypes Can Be Implicated in Dispersal of Endemic and Introduced Begomoviruses in Oman

**DOI:** 10.3390/insects14030268

**Published:** 2023-03-08

**Authors:** Muhammad Shafiq Shahid, Jorge R. Paredes-Montero, Muhammad Ashfaq, Abdullah M. Al-Sadi, Judith K. Brown

**Affiliations:** 1 Department of Plant Sciences, College of Agricultural and Marine Sciences, Sultan Qaboos University, Al-Khod 123, Oman; 2 Department of Biology, Saginaw Valley State University, University Center, Saginaw, MI 48710, USA; 3 Facultad de Ciencias de la Vida, Escuela Superior Politécnica del Litoral (ESPOL), Guayaquil 090605, Ecuador; 4 Centre for Biodiversity Genomics, Department of Integrative Biology, University of Guelph, Guelph, ON N1G 2W1, Canada; 5 School of Plant Sciences, The University of Arizona, Tucson, AZ 85721, USA

**Keywords:** biotype, cryptic species, cytochrome oxidase I, *Geminiviridae*, whitefly vector

## Abstract

**Simple Summary:**

Whiteflies (*Bemisia tabaci*) ‘B mitotype’ represent an insect pest of fruit, vegetable, ornamental and weed plants. *B. tabaci* is a cryptic species comprising at least eight endemic haplotypes, of which haplotypes 6 and/or 8 are recognized invasives. The objective of this study was to identify the prevalence and relationships among putative native and exotic begomoviruses and North Africa–Middle East haplotypes in Oman. Several begomoviral species were identified from *B. tabaci* adults collected from infested plant species, with 67% and 33% representing native and exotic species, respectively. Logistic regression and correspondence analyses predicted ‘strong’ and ‘close’ virus–vector associations involving haplotypes 5 and 2 and the exotic chili leaf curl virus and endemic tomato yellow leaf curl virus-OM, respectively.

**Abstract:**

Irrigated agriculture and global trade expansion have facilitated diversification and spread of begomoviruses (*Geminiviridae*), transmitted by the *Bemisia tabaci* (Gennadius) cryptic species. Oman is situated on major crossroads between Africa and South Asia, where endemic/native and introduced/exotic begomoviruses occur in agroecosystems. The *B. tabaci* ‘B mitotype’ belongs to the North Africa–Middle East (NAFME) cryptic species, comprising at least eight endemic haplotypes, of which haplotypes 6 and/or 8 are recognized invasives. Prevalence and associations among native and exotic begomoviruses and NAFME haplotypes in Oman were investigated. Nine begomoviral species were identified from *B. tabaci* infesting crop or wild plant species, with 67% and 33% representing native and exotic species, respectively. Haplotypes 2, 3, and 5 represented 31%, 3%, and 66% of the *B. tabaci* population, respectively. Logistic regression and correspondence analyses predicted ‘strong’- and ‘close’ virus–vector associations involving haplotypes 5 and 2 and the exotic chili leaf curl virus (ChiLCV) and endemic tomato yellow leaf curl virus-OM, respectively. Patterns favor a hypothesis of relaxed virus–vector specificity between an endemic haplotype and the introduced ChiLCV, whereas the endemic co-evolved TYLCV-OM and haplotype 2 virus–vector relationship was reinforced. Thus, in Oman, at least one native haplotype can facilitate the spread of endemic and introduced begomoviruses.

## 1. Introduction

In Oman, members of the genus *Begomovirus* (family, *Geminiviridae*) have been identified as infecting diverse crops, including vegetables, tropical fruit trees, and wild or uncultivated plant species [1]. The begomoviral genome is ~2.7–2.8 kb in size and consists of a circular single-stranded DNA (ssDNA) encapsulated in a particle, having a twinned, quasi-icosahedral or geminate morphology. Begomoviruses have either a monopartite or bipartite genome, DNA-A, or DNA-A and DNA-B components, respectively. Monopartite begomoviruses occur almost exclusively in the Old World (OW) or Eastern Hemisphere, whereas those extant in the New World (NW) have a bipartite genome, except for the NW monopartite begomovirus tomato leaf curl deformation virus (ToLDeV), found to infect tomato plants in Peru [2].

The whitefly *Bemisia tabaci* Gennadius (Hemiptera: Aleyrodidae) cryptic species group [3,4,5], particularly the B mitotype (or NAFME putative cryptic species), causes damage to crops as a pest and as an insect vector of the genus *Begomovirus*. In Oman, where it is not native, it colonizes wild plant species and agricultural crops, including beans, cucumber, eggplant, mint, okra, pepper, squash, tobacco, tomato, and watermelon, among others. Begomoviruses are transmitted by *B. tabaci* in a circulative, non-propagative manner [3]. Studies have shown that the extent of transmission specificity and, subsequently, transmission efficiency or ‘competency’ may be most optimal among phylogeographically congruent begomoviral species–whitefly cryptic species combinations [6,7,8,9]. However, several studies have suggested that certain *B. tabaci* cryptic or specific haplotypes may be promiscuous in that they may be capable of transmitting one or more begomoviruses with which they have not co-evolved [3,6,10,11,12]. Thus, predicting compatible and incompatible whitefly vector–begomovirus combinations is difficult, despite the overall increased knowledge about whitefly–virion protein–protein interactions that govern viral transmission and of the phylo-biogeographical distribution and microclimate niche affiliations of whitefly–begomovirus combinations, which may collectively define boundaries or give rise to determinants of specificity (phenotypes) [13].

Differentiation of *B. tabaci* populations at a finer level, such as by microsatellites mitotypes (*mtCOI*) [3] and haplotypes (*mtCOI* SNPs), have been used to resolve information on co-evolutionary lineages and associated begomoviral populations, strains, and species. In a recent study, the *B. tabaci mtCOI* sequence was shown to be evolving 12.5 times faster than the nuclear genome [4], making it a valuable marker for discerning micro-evolutionary scale changes. The availability of the *mtCOI* sequence for a large sample size of the B mitotype/cryptic species has facilitated the identification of eight SNPs haplotypes of the ‘B mitotype’ or NAFME cryptic species, NAFME 1–8, which have either strict or overlapping geographical affinities as well as microclimate niches within. The NAFME 6 and 8 are now recognizable as the pervasive, exotic *B. tabaci* haplotypes that have invaded locales in Asia, Africa, Australia, Europe, the Mediterranean region, and the New World [14]. Haplotypes NAFME 1–3 co-exist in the desert regions of the Arabian Peninsula. The prototype NAFME 3 field isolate was first identified in Ethiopia in the 1990s. The sister haplotypes NAFME 4 and 5 are predominant in the semi-arid niches of Iran and Pakistan. Haplotypes NAFME 6–8 appear to be adapted to both the Mediterranean and desert climates found in Israel and Egypt, leading to the hypothesis that NAFME 6 dispersed/spread eastward to Asia, while NAFME 8 spread westward and colonized the New World and elsewhere [14]. Finally, in a previous study, haplotype NAFME 5 was identified in Nizwa, Oman, thus far the only location where it has been documented [14]. Additional sampling is expected to reveal whether NAFME 1–3 haplotypes may also occur here, which would be consistent with macroclimate predictions of the NAFME 1–3 endemism.

The B mitotype and its haplotypes [14] and many previously described monopartite begomoviruses are endemic to the area encompassing northeastern Africa (adjacent to the Red Sea), the Arabian Peninsula, and southwestern Asia [3]. Transmission studies with China-native or recently introduced B mitotypes and *Cotton leaf curl Multan virus* (CLCuMuV-Fai), introduced from the Indian subcontinent to China, have shown that CLCuMuV-Fai is transmissible by a laboratory-maintained colony of an Asia II 1 mitotype, which is native to parts of both China and the Indian subcontinent. However, colonies of a China-native Asia 1 mitotype and of the non-native B and Q mitotypes prevalent in agroecosystems in China did not transmit the introduced CLCuMuV-Fai isolate [15,16,17,18,19]. These and other reports of less-than-strict begomovirus-vector specificity between endemic–non-endemic virus–vector combinations [6,12,20,21] have challenged the paradigm that *B. tabaci*–begomovirus relationships do not necessarily coincide with co-endemism, as has been previously hypothesized [13]. Thus, understanding the extent to which begomovirus-*B. tabaci* mitotype and/or haplotype transmission specificity has evolved, with a strict or relaxed basis in co-endemism, could greatly inform the risk assessment of exotic and non-exotic virus–vector combinations with small- or global-scale invasion potential.

The objective of this study is to determine whether the distribution of exotic (introduced) and native whitefly *B. tabaci* haplotypes in Oman are associated with extant native or introduced begomoviral species. Plants exhibiting symptoms reminiscent of begomovirus infection and *B. tabaci* whiteflies infesting symptomatic plants were collected from different governorates in Oman during the growing seasons and analyzed for begomovirus presence. Begomoviruses were provisionally identified by sequence analysis of a 688-base pair (bp) fragment of the begomoviral *cp*. Identification of whitefly *B. tabaci* haplotypes was based on informative *mtCOI* SNPs previously shown to differentiate the eight known haplotypes associated with the NAFME cryptic species [14]. A correspondence analysis (CA) and logistic regression were carried out to identify potential associations between the occurrence of whitefly haplotype and begomoviral species.

## 2. Material and Methods

### 2.1. Whitefly and Plant Field Collections

Adult whiteflies were collected from whitefly-infested crop plants and weed species during 2015–2018 from 34 sites in seven Oman Governorates. Multiple samples were collected from symptomatic cultivated or uncultivated (wild) host plants exhibiting leaf curling, vein thickening, and/or enations, indicative of virus-like diseases. Five to ten whitefly adults were collected from infested plants, approximately ~500 feet apart, per collection site. Each whitefly sample consisted of a minimum of five adults infesting the leaves of an infested plant. Whiteflies were collected live using a hand-held aspirator, transferred to a 1.5-mL microfuge tube containing 95% alcohol, and stored at −20 °C (Table 1). For each sample, the collector’s name, date of collection, and field location GPS coordinates were recorded (Table 1). For plant samples, leaf samples were collected from the growing tip of symptomatic vegetable, ornamental, or wild (uncultivated) plants (n = 36), exhibiting begomovirus-like symptoms consisting of leaf curling, mosaic, or mottling, shortened internodes, and overall stunting. Leaf samples collected from each symptomatic plant were placed in a separate plastic bag, transferred to an insulated container with ice, transported to the laboratory, and stored at −4 °C (Table 1).

### 2.2. Total DNA Isolation from Plants and Whiteflies, PCR Amplification, DNA Sequencing, and Sequence Analysis

Total nucleic acids were isolated from leaves collected from symptomatic plant samples and from individual whiteflies using the E.Z.N.A.^®^ Insect DNA Kit (Omega Bio-tek, Norcross, GA, USA) for plant tissue and whiteflies, respectively, a commercial kit based on the CTAB method [22].

### 2.3. Begomoviral Coat Protein Gene Fragment

The begomoviral coat protein fragment was amplified from total genomic DNA. A 688-base pair (bp) fragment of the begomoviral species-informative region of the coat protein (‘core cp’) was obtained by PCR amplification from DNA purified from the plant using the primers AV1-F/R (5′-ATCATTTCCACKCCCGYCTCGA-3′/5′-GCRTGMGTACABGCCATATACA-3′) [23]. The AV1 fragment is nested within the coat protein region (cp) of the begomovirus and has been validated extensively as a phylogenetically informative sequence for tentative begomovirus species identification. Cycling parameters were denaturation at 93 °C for 30 s, followed by 30 cycles of 93 °C for 30 s, 55 °C for 30 s, 72 °C for 40 s, and a final extension at 72 °C for 10 min. The PCR product size was estimated by agarose gel (1.5%) electrophoresis (TAE buffer, pH 8.0), and visualized by staining with ethidium bromide (Thermo Fisher Scientific, CA, USA). The PCR amplicons were cloned into the pTZ57R/T plasmid vector (Thermo Fisher Scientific, CA, USA). The plasmids containing an insert of the expected size were confirmed by restriction enzyme analysis and inserts of the expected size were sequenced completely by Sanger DNA sequencing (Macrogen Inc.; Seoul, Republic of Korea).

Preliminary begomovirus identification was carried out by phylogenetic analyses and by subjecting core *cp* fragments to a BLASTn [24] search of the NCBI, using a virus reference sequence database to identify the top hits. Sequences representing the top twenty hits with the highest similarity score, at 99–100%, coverage (95–100%), and e-values of less than 10^−2^ were downloaded from the GenBank database. Downloaded references (accessed on 16 March 2021) were used to build phylogenetic trees. Phylogenetic analysis was carried out using the Bayesian algorithm [25], and the tree was drawn with TreeAnnotator v1.84 [26] and visualized with FigTree v1.42 (http://tree.bio.ed.ac.uk/software/figtree/, accessed on 1 March 2023).

Pairwise distances for the core *cp* sequences were calculated using the Sequence Demarcation Tools (SDT) software v1. 2 [27], and a cut-off of <88–91% was used to assign the provisional species names [28]. Previous calibrations of *core cp* region divergence over the last twenty years (authors, unpublished results) indicated that pairwise distance comparisons of the core *cp* with the respective complete genome sequence were only slightly higher and did not result in discrepancies in species predictions, except those with predicted recombination within in the vicinity of the coat protein and/or the *cp* as well as leftward or rightward sequences that could be documented. Here, the phylogenetic analysis prediction (provisional core *cp*) of species placements on the tree was consistent with the results of BLASTn (GenBank virus Refseq) and pairwise distances (SDT) analyses.

### 2.4. Whitefly Mitochondria Cytochrome Oxidase I Fragment

Each adult whitefly was ground individually with a micropipette-melted tip and homogenized in 350 μL CTL Buffer and 25 μL Proteinase K Solution (50–100 µg/mL) in a 1.5 mL microcentrifuge tube, followed by incubation at 60 °C for 30 min. An equal volume of chloroform:isoamyl alcohol (24:1) was added, and the solution was vortexed and centrifuged (Eppendorf 5452 Mini Spin Centrifuge) at 10,000× *g* for 3 min. The supernatant was mixed with one volume of BL Buffer (Omega Bio-tek, Inc., Norcross, GA, USA) and 2 μL RNase A, followed by incubation at 70 °C for 10 min. The total DNA preparation was column-purified using a HiBind^®^ DNA Mini Column (Omega Bio-tek, Inc., Norcross, GA, USA). The HiBind^®^ DNA Mini Column was washed with HBC and DNA wash buffers according to the manufacturer’s instructions. The total purified DNA was eluted in 10 mM-Tris-EDTA buffer and stored at −20 °C.

The 3′-end fragment of the *mtCOI* gene was PCR-amplified using primers (C1-J-2195 5′–TTGATTTTTTGGTCATCCAGAAGT–3′; TL2-N-3014 5′–TCCAATGCACTAATCTGCCATATTA–3′) [29] to obtain an expected size product of 865 bp. Cycling conditions were as follows: denaturation at 93 °C for 30 s, followed by 35 cycles of 93 °C for 30 s, 52 °C for 30 s, 72 °C for 45 s, and a final extension at 72 °C for 10 min. At least two amplicons per sample of the expected size were cloned into the pTZ57R/T plasmid vector (Thermo Fisher Scientific, California, USA). Plasmids containing the expected size of the insert were confirmed by restriction digestion. The confirmed cloned inserts were sequenced (Sanger) bi-directionally (Macrogen Inc.; Seoul, Republic of Korea).

### 2.5. SNPs Classification of Haplotypes of North African–Middle East B Mitotype

The *mtCOI* sequences were screened to identify informative single nucleotide polymorphisms (SNPs) [14] in Geneious Prime v2021.1.1 using the ‘Find Variants’ tool, using 2% as the minimum variant frequency. The Arizona B biotype prototype (GenBank accession AY057123) was included as the reference sequence. The SNP profiles were compared to a recently published global alignment of NAFME haplotypes [14] that groups the NAFME (B) haplotypes into eight NAFME clades by phylogenetic analysis using a Maximum Likelihood (ML) algorithm. The RaxML phylogenetic tree was constructed using 100 bootstrap iterations, optimized substitution rates, and the GAMMA model of rate heterogeneity. The *B. tabaci* putative MEAM2, which is recognized as a pseudogene (GenBank accession MT605827), was used to root the tree.

### 2.6. Spatial Distribution of Bemisia Tabaci NAFME Haplotypes

The geographic distribution map of the NAFME haplotypes identified within Oman and reference sequences determined for other locations in the Middle East were drawn using the geographic coordinates for each sample. Maps of administrative divisions were downloaded from the online geographic information systems platform DIVA-GIS (https://www.diva-gis.org/, accessed on 1 March 2023), and occurrence records of NAFME haplotypes were compiled in QGIS v3.8.1 (QGIS development team, 2021) using the ‘Biological Records Tool’ plugin.

### 2.7. Correlation and Correspondence Analyses of Whitefly Vector–Begomovirus Associations

The significance of associations between whitefly haplotypes and begomovirus species detected in whitefly and plant tissues were tested using a logistic regression approach using the ‘glm’ function of the ‘stats’ package v4.2.0 (R Core Team 2021). The PCR amplification results were entered into a text matrix as a binomial variable, where 1 and 0 corresponded to the presence and absence of a begomoviral species, respectively. The begomovirus species identified in adult whiteflies and plant samples were evaluated to determine if potentially significant correlations existed between the occurrence and identity of the whitefly vector haplotype, except when a begomoviral species was detected in a single plant or whitefly sample. The correspondence analysis was carried out using the software InfoStat v.2020 [30] by tabulating a contingency table, with Euclidean distances assigned to each pair of categories within each discrete variable under study. Each pair of categories was drawn in a biplot (two-dimensional plot) on two axes coordinates that define the Euclidean distance between them [31]. The variance explained by one of two axes of the biplot was given by the ‘eigenvalues’, represented as the proportion of the contribution of each axis that explained the total variability of the data.

## 3. Results

### 3.1. Classification of Whitefly Haplotypes Based on SNPs and Phylogeographical Relationships

**Figure 1:**
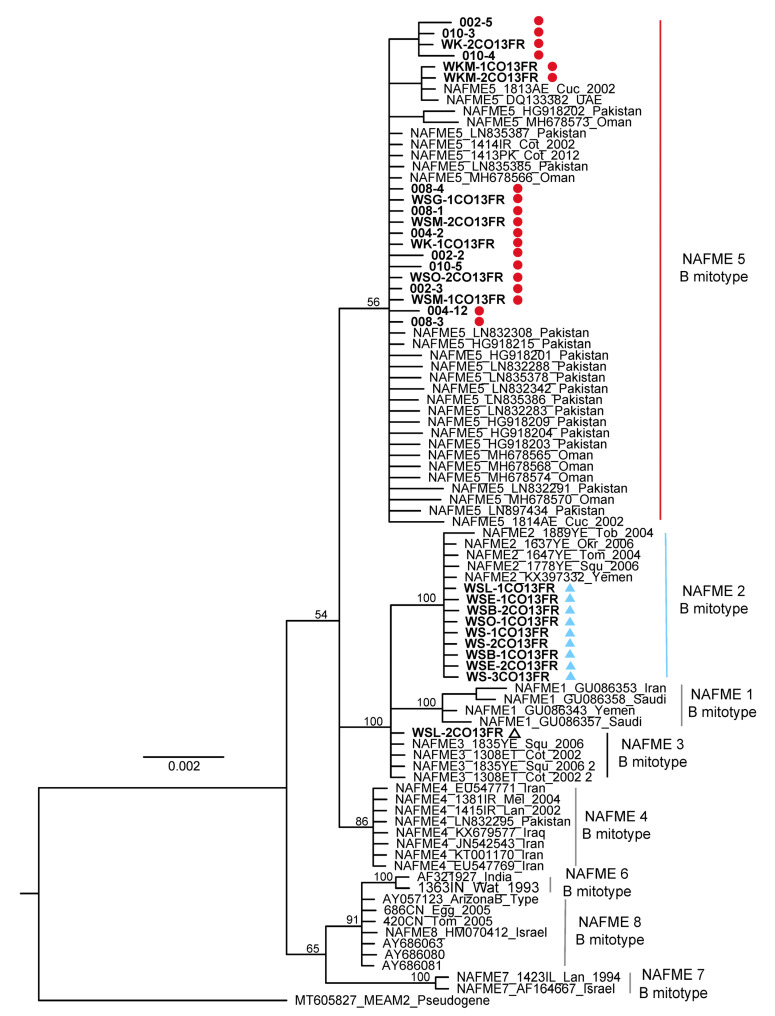
Maximum likelihood tree for mitochondrial cytochrome oxidase I gene sequences of the NAFME haplotype groups, reconstructed using RaxML. Whitefly haplotypes NAFME 2, 3, and 5 grouped most closely with reference sequences belonging to the North Africa–Middle East (NAFME) clade. One thousand bootstrap iterations were carried out, and statistical support values (>60%) were placed at the major nodes.

The 34 *mtCOI* sequences determined in this study were differentiated as haplotypes based on one to eight SNPs identified in the 3′-end of the COI gene [14]. The AY057123 GenBank sequence was used as the reference *mtCOI* sequence for SNP identification. The AY057123 sequence corresponds to the prototype AZ-B (previously, ‘biotype’) (Brown Lab, UA Tucson, Arizona), recognized as an invasive *B. tabaci* introduced in the US in approximately 1988–1989 [3]. The predominant SNP profile corresponded to haplotype NAFME 5 and revealed four nucleotide transitions, one thiamine, and three cytosine residues at positions 826, 1137, 1152, and 1375, respectively. NAFME 2 was characterized by having four fixed cytosine residues at positions 810, 1068, 1137, and 1152, respectively, a transition from a guanine to an adenine residue at position 1053, and a transversion from a thymine to a guanine residue at position 1074. The NAFME 3 haplotype shared fixed nucleotides with NAFME 2 at positions 1068, 1074, 1137, and 1152 of the *mtCOI* gene [16] (Supplementary Figure S1). No whiteflies analyzed from the Oman collections belonged to two recently recognized invasive haplotypes, NAFME 6 or 8, of which only NAFME 8 has a near-cosmopolitan distribution on several continents. Similarly, the phylogenetic (ML) analysis resolved the whitefly haplotypes from Oman into one of the three NAFME clades 2, 3, and 5 in relation to previously published reference sequences (Figure 1 and Supplementary Figure S1) [14].

**Figure 2:**
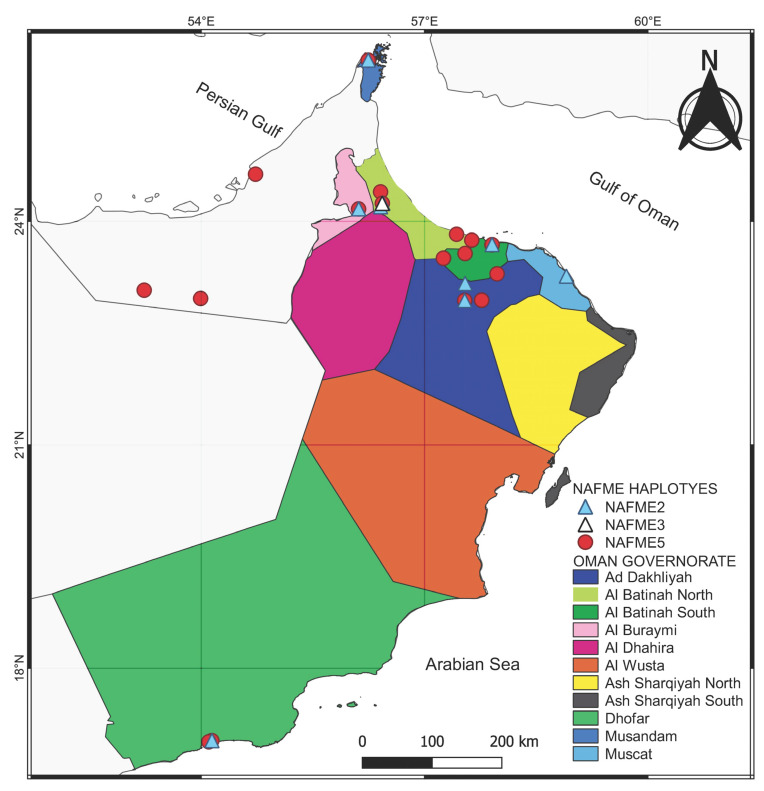
The distribution of the *Bemisia tabaci* belonging to the North Africa–Middle East (NAFME) cryptic species in Oman.

The NAFME 2 and 5 haplotype distributions overlap geographically, with both inhabiting the northern and southern regions of Oman. However, NAFME 5 was by far the most abundant haplotype, followed by NAFME 2, at 66 and 31%, respectively. Among the *B. tabaci* whiteflies collected in this study, only the collection from Al-Batinah North was identified as NAFME 3 (Figure 1 and Figure 2).

### 3.2. Provisional Begomovirus Identification and Species Associated with NAFME Haplotypes

Of thirty-six collected whitefly samples, sixteen confirm the presence of nine different begomovirus species ((Supplementary Figure S2 and Table 1). These begomoviruses, considered to be native or to have originated from Oman and surrounding locales, were *Tomato leaf curl Barka virus* (ToLCBrV), *Tomato leaf curl Liwa virus* (ToLCLwV), *Tomato yellow leaf curl virus-Oman* (TYLCV-OM), and *Watermelon chlorotic stunt virus* (WmCSV), while the exotic viruses known to be introduced from Africa are *Cotton leaf curl Gezira virus* (CLCuGeV) and *Tomato leaf curl Sudan virus* (ToLCSDV) from Asia, with *Chili leaf curl virus* (ChiLCV), *Mungbean yellow mosaic virus* (MYMIV), and *Squash leaf curl virus* (SLCV) having their origins in the Americas (New World). We detected ChiLCV, ToLCBrV, ToLCLwV, and TYLCV-OM begomovirus species from whitefly haplotypes. Among these, the begomovirus detection rate was highest for ChiLCV (50%), followed by TYLCV-OM (37.5%), whereas ToLCBrV and ToLCLwV were each detected at a frequency of 6.25%.

**Figure 3:**
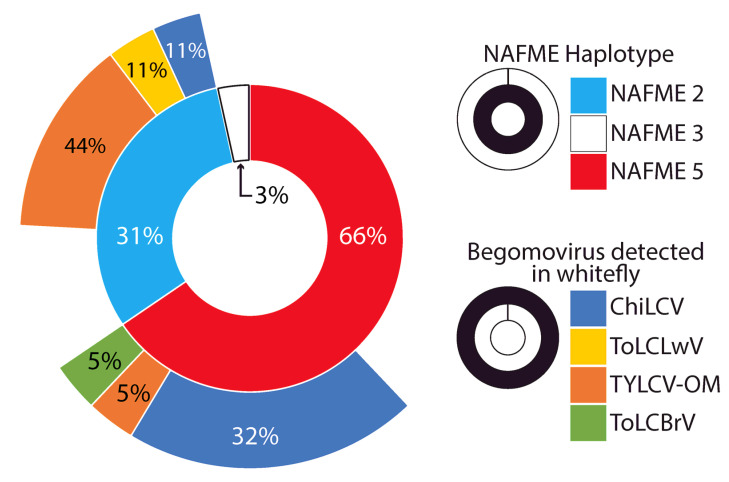
Frequency of *Bemisia tabaci* NAFME haplotypes (inner chart layer) and begomoviral species (outer chart layer) detected in whitefly extracts.

NAFME 2 showed the largest rate of virus detection at 66.7%, followed by 42.1% for NAFME 5 (Figure 3). Unexpectedly, begomoviral amplicons were not detected for the *B. tabaci* NAFME 3 haplotype. The detection rate of ChiLCV for NAFME 5 and NAFME 2 was 31.6% and 11%, respectively. The detection rate of TYLCV-OM was 44.4% and 5% for NAFME 2 and NAFME 5, respectively. Additionally, ToLCLwV and ToLCBrV were associated with the NAFME 2 haplotype at an 11% detection rate each (Figure 3). The geographic distribution of ChiLCV and TYLCV-OM was found to be widespread; however, they varied in prevalence and by location within the five Governorates of Al-Batinah, Al-Musandam, Dhofar, Ad-Dakhilya, and Ash Sarkyah. In contrast, the begomoviral species, CLCuGeV, MYMIV, ToLCBrV, ToLCLwV, ToLCSDV, and WmCSCV, were detected in *B. tabaci*-infested host plants in the mainland Al-Batinah Governorate only and were associated with either NAFME 5 and NAFME 2 haplotypes at low detection rates of 11% or below.

*B. tabaci* whiteflies were collected from host species from different agroecosystems, including vegetables grown in greenhouses and home gardens, field crops, fruit crops, and wild, uncultivated plant species (Table 1). The NAFME 2 haplotype was the predominant *B. tabaci* in the Dhofar Governorate, which is located about 1000 km from the main agriculture area in Al-Batinah. Haplotype 2 was associated with begomovirus-infected cucurbit species, mint, and tomato plants (Table 1). The NAFME 5 haplotype occurred throughout Oman and colonized begomovirus-infected papaya, pepper, tobacco, tomato, watermelon, and wild (uncultivated) host species. The NAFME 3 haplotype was detected in the Musandam governorate, where it occurred on pumpkin plants, a species from which ChiLCV has been detected in other collection sites, albeit not in this location (Table 1).

Nine begomovirus species were provisionally identified based on PCR amplification of the core coat protein gene from *B. tabaci* adults. Most of the begomoviruses detected were associated with *B. tabaci*-infested vegetable crop species, including cucumber, mint, pepper, pumpkin, tobacco, tomato, and watermelon. Begomoviruses were also identified in papaya trees and several weeds. The non-native ChiLCV was identified in *B. tabaci* collected from papaya, pepper, mint, tobacco, tomato, and an unidentified species of *Urtica*. Whiteflies collected from cucumber, papaya, pumpkin, and tomato were shown to harbor the native begomovirus TYLCV-OM, while whiteflies collected from tomato plants were infected with two endemic begomoviruses, ToLCVBrV and ToLCLwV. However, no begomoviral species were amplified by PCR from NAFME 3 whiteflies collected in the Musandam governorate (Table 1).

### 3.3. Associations between NAFME Haplotypes and Begomoviruses

**Figure 4:**
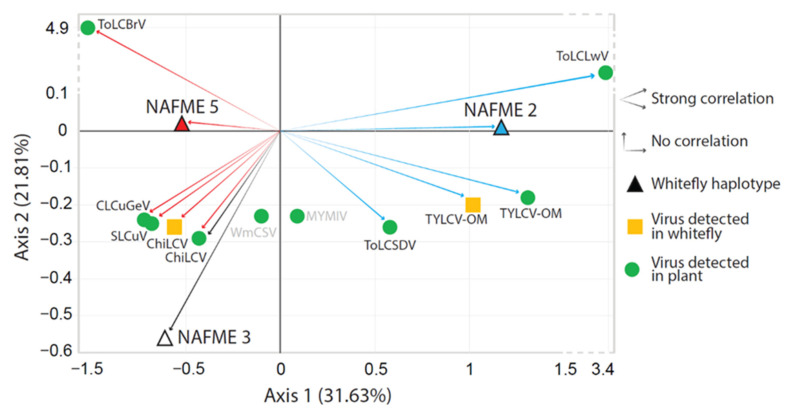
Correspondence analyses between whitefly haplotypes NAFME 2, 3, and 5 and begomovirus species detected in whitefly and plant samples. Red arrows show positive associations between begomovirus species and whitefly haplotype NAFME 5; blue arrow indicates positive associations between begomovirus species and NAFME 2 haplotype. Black arrow shows the association between the single begomovirus species detected in symptomatic plants infested by the NAFME 3 haplotype.

A linear regression model was implemented using the logistic regression method to assess the significance of putative associations between the whitefly haplotypes and begomovirus (es) detected in plants and whitefly adults. The logistic regression coefficients indicated that the probability of NAFME 5 harboring TYLCV-OM was significantly decreased compared to the probability of NAFME 2 harboring the same begomovirus species (*p* = 0.0186), indicating that a strong association was supported between TYLCV-OM and the NAFME 2 haplotype. In contrast, the probability of NAFME 2 harboring ChiLCV was significantly decreased compared to the probability of an association with NAFME 5 (*p* = 0.0499), suggesting a strong correlation between haplotype 5 and ChiLCV prevalence. Correspondence analysis, with 53% of total variance explained, was consistent with the logistic regression test results, predicting a strong association between TYLCV-OM and ChiLCV and NAFME 2 and 5, respectively. For begomoviral species detected/identified in whitefly-infested plants, the correspondence analysis results indicated a positive association (*p* < 0.05) between NAFME 2 and the begomoviral species ToLCLwV, ToLCSDV, and TYLCV-OM. Similarly, ChiLCV, CLCuGeV, SLCuV, and ToLCBrV were strongly associated (*p* < 0.05) with the NAFME 5 haplotype collections (Figure 4). Positive associations were not observed (unsupported) for MYMIV and WmCSV and NAFME haplotypes 1–3.

## 4. Discussion

### 4.1. Classification of Whitefly Haplotypes Based on SNPS and Phylogeographical Relationships

The NAFME cryptic species [4], also referred to as the ‘B mitotype’ based on *mtCOI* typing [3,32] and, previously, as MEAMI cryptic species [33], comprises at least eight phylogeographic haplotype groups, designated as NAFME 1–8 [14]. Only two of these haplotypes, NAFME 6 and 8, have become invasive (e.g., haplotype 8 in the Americas and haplotype(s) 6 and/or 8 in China/Asia), establishing themselves in locales distant from their center of origin in the North Africa–Middle East region (NAFME) [14]. The distribution of the other six haplotypes, their vector competence, and interactions in their native habitats have remained poorly studied until recently. In this study, a country-wide sampling of whiteflies and associated plant tissues was conducted in agroecosystems in Oman to better understand the association between native NAFME haplotypes and the indigenous or exotic begomovirus species they transmit in the desert regions of the Arabian Peninsula, one of three major macro-niches that native NAFME haplotypes inhabit [14].

Based on these results, three NAFME haplotype groups (MEAMI species) were differentiated based on the unique SNPs that correlate to the haplotypes previously identified in the Middle East—NAFME 2, 3, and 5, with NAFME 5 being the predominant haplotype, at a frequency of 66% of the total samples analyzed. Previous studies have shown that NAFME 5 is also the predominant haplotype in Iran and southern Pakistan, suggesting its region of endemism is characterized by the semi-arid climates of the Irano-Turanian region and, potentially, one or more fragmented microclimate niches in the southern deserts of Pakistan [14]. Haplotypes NAFME 2 and 5 are known to occur in the Dhofar Governorate, near the Yemen border in southern Oman, and 1000 km away, in the Al-Batinah, the main agriculture production area in Oman. Additionally, these two NAFME haplotypes have been identified in Khasab in the Al-Musandam Governorate, northwards and near the border of the UAE, 500 and 1500 km from the Al-Batinah and Dhofar regions, respectively. Further, NAFME 2 and 5 overlap in Salalah, where a unique topography is created by the Al-Hajar mountain range that divides the country into northern and southern Oman. The range of NAFME 2 and 5 was found to overlap, despite the low frequency of NAFME 2, at 31%. Additional sampling will be required to determine if the less than 50:50 representation of the two haplotypes might indicate the impending displacement of NAFME 2 by NAFME 5. Such widespread distribution of NAFME 2 and 5 haplotypes in Oman may be the result of ethological variables at play, including but not limited to their flight behavior. In laboratory studies, the B mitotype (now recognized as haplotype NAFME 8) of *B. tabaci* has been demonstrated to utilize primarily ‘trivial flight’ or engage in relatively short-distance flights between plant hosts; however, trapping studies conducted under field conditions have shown they also exhibit migratory behavior, dispersing long distances on low winds (~20 ft above the landscape), as far as 2.7 km and much further over time [34,35,36,37].

The NAFME 3 haplotype of *B. tabaci* was identified only in Al-Batinah (north), suggesting either a highly restricted distribution and, potentially, unique microclimate characteristics [38] and/or that future collections or surveys would benefit from a greater sample size to determine the factors that limit the distribution of this *B. tabaci* haplotype, which is apparently rare in Oman agroecosystems. Previous studies have demonstrated and/or inferred the propensity for phenotypic differences within and among *B. tabaci* and its cryptic species [3]. Among the recognized phenotypic differences are host range, microclimatic factors, and, among the haplotypes in Oman, potential resource competition between NAFME 2 and 5. The broad and/or limited distribution of *B. tabaci* mitotypes has been documented in the Indian subcontinent for selected mitotypes in Pakistan, Asia II 1, 7, and 5, and for the A and B mitotypes in the American Tropics. In this study, the two haplotypes documented at the lowest frequency, NAFME 2 and NAFME 3, were collected from vegetation in the urban landscape and household gardens rather than in monoculture cropping systems. This pattern is consistent with observations of native *B. tabaci* mitotypes in Ecuador and Pakistan, respectively [39]. In this study, the NAFME 5 haplotype colonized a broad range of plant host species/families, which is a previously recognized phenotype of the invasive NAFME 8; for at least some well-studied *B. tabaci*, albeit in previous historical surveys, the cryptic species of *B. tabaci* could not yet be ascertained [40].

### 4.2. Provisional Begomovirus Identification and Species Associated with NAFME Haplotypes

The results of the survey designed to detect begomoviral prevalence in plant host(s) and in the prospective whitefly vector haplotypes of *B. tabaci* indicated an association of ChiLCV, CLCuGeV, MYMIV, SLCV, ToLCBrV, ToLCLwV, TYLCV-OM, ToLCVSDV, and WmCSV, which were identified from plant hosts, whereas four of them, ChiLCV, ToLCBrV, TYLCV-OM, and ToLCLwV were detected only in whiteflies.

The nine monopartite and/or bipartite begomoviruses identified in this study were found to infect one or more plant hosts, primarily vegetable crops, cucumber, eggplant, mungbean, muskmelon, okra, pepper, pumpkin, tobacco, tomato, and watermelon; two ornamentals–herbs: mint (*Lamiaceae*) and *Senna* spp. (*Fabaceae*); an annual tropical fruit, papaya; and an uncultivated species, *Urtica* spp. (*Urticaceae*), collectively (Table 1), representing seven plant families. In Oman, at least fourteen begomoviral species (monopartite and bipartite), including those provisionally identified herein, have been reported previously from vegetable crops, including the monopartite ChiLCV, *Chickpea chlorotic dwarf virus* (CpCDV), CLCuGeV, *Okra leaf curl Oman virus* (OLCOMV), ToLCABV, ToLCBrV, ToLCLwV, ToLCOMV, ToLCSDV, the bipartite *East African cassava mosaic Zanzibar virus* (EACMZV), MYMIV, SLCV, *Tomato leaf curl Palampur virus* (ToLCPlV), and WmCSV [1,41,42,43,44,45].

### 4.3. Associations between NAFME Haplotypes and Begomoviruses

Based on logistic regression and correspondence analyses, the various begomovirus-host plant–whitefly haplotype combinations were not equally associated with the two most prominent *B. tabaci* haplotypes, NAFME 2 and 5. Instead, ChiLCV, of Indian subcontinent endemism, was ‘strongly associated’ (78%) with NAFME 5, a haplotype predicted to be native to Iran and, also possibly, Pakistan, whereas the *B. tabaci* haplotype NAFME 2 was ‘strongly associated’ (72%) with the type TYLCV-OM strain, extant in Oman.

Among the collection sites in Oman, the NAFME 5 haplotype colonized many species of plants and was associated with as many as nine begomoviral species detected in the plant host or putative whitefly vector (Table 1). These observations predict that *B. tabaci* haplotype 5 harbors transmission competency for begomovirus species extant in Oman, with which it has and has not co-evolved. Similarly, the NAFME 2 haplotype was predicted to exhibit a strong association with two native (endemic) begomoviral species, TYLCV-OM and ToLCLwV, with which it is known to have co-evolved. The scenario for TYLCV-OM was more complex than for other begomoviruses identified in Oman agroecosystems, primarily because the geographical region (extant), comprising Iran and the Arabian Peninsula, is the recognized ancestral center of diversification of all strains and species of the TYLCV group, of which five are extant in Iran, including TYLCV-OM [44], whereas the TYLCV species group representation in Oman is limited to TYLCV-OM. The genome sequence of several begomoviruses extant in Oman, including the predominant and widespread TYLCV-OM species, exhibit extensive genetic variability, indicating ongoing diversification in Iran and Oman, likely due to geographic isolation. Indeed, the distribution of the haplotype NAFME 5, which is native to a unique microclimate niche occurring in Iran and Pakistan, respectively [14], suggests that NAFME 5 has potentially co-evolved with all the seven recognized TYLCV species and strains [44], albeit, in Oman, the NAFME 5 haplotype was not preferentially strongly associated with TYLCV-OM. Notably, TYLCV and its seven recognized species/strains have not been reported to occur in Pakistan, despite the occurrence of the NAFME 5 haplotype in the deserts of southern Pakistan, located in Bahawalpur and Sindh, respectively, regions of sparse agriculture [21]. However, the potential for the occurrence of TYLCV-OM in south Pakistan cannot be ruled out, given the recent report of the emergence of a new TYLCV-OM species identified from cluster bean (guar) and tomato, *Tomato leaf curl Oman virus* (ToLCOMV-PK, previously, TYLCV-PK) and its associated tomato leaf curl betasatellite, ToLCB, a predicted recombinant between TYLCV-OM and *Papaya leaf curl virus* (PaLCV) [45,46], the latter betasatellite, which is considered native to the Indian subcontinent.

The results of the logistic regression and correspondence analysis also predicted a ‘strong association’ between NAFME 5 and exotic/introduced begomoviruses prevalent in plant hosts in Oman (*p* = 0.0499). The latter introduced viruses, CLCuGeV, MYMIV, SLCV, and WmCSV, are endemic to Asia, the Middle East, Africa, and the Americas, respectively. There is knowledge and evidence of their transmission in Oman by the predicted, non-native NAFME 5 *B. tabaci* [1,47,48] and in other locations worldwide, where it has been established only recently following its introduction [3]. This scenario does not support the paradigm that *B. tabaci* cryptic species vector competency and a predicted co-evolution with begomoviral species are linked.

Collectively, these observations suggest that the evolution of vector competence may not be strictly determined by long-standing co-evolutionary forces but rather shorter and near-term adaptation, in line with a set of specific environmental conditions and/or repeated exposures, resulting in promiscuous evolutionary or ‘relaxed’ co-evolutionary outcomes that promote survival and continued diversification. Understanding the basis for begomovirus–whitefly transmission specificity, evolved under strict or relaxed conditions, is paramount to identifying the mechanisms underlying whitefly vector transmission specificity and subsequent competency. This, together with precise environmental and phylo-biogeographical data, importantly, could aid in predicting the begomovirus-*B. tabaci* cryptic species combinations are most likely to result in locally restricted or long-distance dispersal events, with the potential to lead to outbreaks and/or widespread epidemics.

## Figures and Tables

**Table 1:** Sample number, year of collection, location of collection with GPS coordinates, host plant, agroecosystem, provisional begomoviral species associated with plant and whitefly samples, begomoviral predicted center(s) of diversification, GenBank Accession number for begomoviral and mitochondrial cytochrome oxidase I (COI) sequence, and *Bemisia tabaci* haplotype based on SNPs analysis.

Field sample Identification Number	Collection Year	Location/ GPS Coord.	Plant Host and Family	Agroecosystem	Plant-Infecting Begomo- Virus *	Begomovirus Predicted Center of Origin	Whitefly-Associated Begomovirus	Begomoviral Core Coat Protein Accession Number	Cytochrome Oxidase I Gene Accession Number	*Bemisia tabaci* Haplotype Identification by SNPs
004-12	2015	Al Barka/ 23.6837° N 57.9049° E	Tomato *Solanaceae*	Crop	CLCuGeV/ChiLCV	North Africa/Indian subcontinent	ChiLCV	OM326816	OM368461	NAFME5
WSB-1CO13FR	2015	Al Qurayyat/ 23.2652° N 58.9034° E	Tomato *Solanaceae*	Crop	ChiLCV	Indian subcontinent	No begomovirus detected	NA **	OM368471	NAFME5
010-5	2015	Al-Batinah/ 23.5684° N 57.5432° E	Mungbean *Fabaceae*	Crop	MYMIV	Indian subcontinent	No begomovirus detected	NA	OM368460	NAFME5
002-5	2015	Al Barka/ 23.6857° N 57.9048° E	Watermelon *Cucurbitaceae*	Crop	ChiLCV	Indian subcontinent	ChiLCV	OM326817	OM368454	NAFME5
005-1HamFR	2015	Al Qurayyat/ 23.2652° N 58.9034° E	Cucumber *Cucurbitaceae*	Crop	TYLCV-OM	Mediterranean and/or Middle East	No begomovirus detected	NA	OM368451	NAFME5
008-1_28FR	2016	Al Barka/ 23.6837° N 57.9049° E	Tomato *Solanaceae*	Crop	ToLCLwV	Middle East	ToLCLwV	OM326823	OM368473	NAFME2
WSE-2CO13FR	2016	Salalah/ 17.0302° N 54.1435° E	Mint *Lamiaceae*	Home garden	ChiLCV	Indian subcontinent	ChiLCV	OM326818	OM368468	NAFME2
008-2_28FR	2016	Al Buraymi/ 24.1679° N 56.1149° E	Tomato *Solanaceae*	Crop	TYLCV-OM	Mediterranean and/or Middle East	TYLCV-OM	OM326813	OM368463	NAFME2
WS-1CO13FR	2016	Al Barka/ 23.6878° N 57.9059° E	Tomato *Solanaceae*	Crop	MYMIV/TYLCV-OM	Indian subcontinent/Middle East	TYLCV-OM	OM326812	OM368475	NAFME2
010-4	2016	Khasab/ 26.1644° N 56.2426° E	*Senna* spp. *Fabaceae*	Weed	ChiLCV	Indian subcontinent	No begomovirus detected	NA	OM368450	NAFME5
008-1	2016	Salalah/ 17.0302° N 54.1415° E	Eggplant *Solanaceae*	Home garden	No begomovirus detected	NA	No begomovirus detected	NA	OM368465	NAFME5
002-3	2016	Khasab/ 26.1654° N 56.2426° E	Tomato *Solanaceae*	Crop	CLCuGeV	North Africa	No begomovirus detected	NA	OM368452	NAFME5
WSB-2CO13FR	2016	Al Buraymi/ 24.1679° N 56.1149° E	Tomato *Solanaceae*	Crop	TYLCV—OM	Mediterranean and/or Middle East	TYLCV-OM	OM326811	OM368473	NAFME2
WSL-1CO13FR	2016	Khasab/ 26.1664° N 56.2432° E	Muskmelon *Cucurbitaceae*	Crop	No begomovirus detected	NA	No begomovirus detected	NA	OM368472	NAFME2
WSO-2CO13FR	2016	Sohar/ 24.396° N 56.408° E	Tobacco *Solanaceae*	Crop	ChiLCV	Indian subcontinent	ChiLCV	OM326819	OM368463	NAFME5
WK-1CO13FR	2016	Khasab/ 26.1654° N 56.2432° E	Squash *Cucurbitaceae*	Crop	SLCuV	Central and North America	No begomovirus detected	NA	OM368467	NAFME5
WKM-1CO13FR	2016	Salalah/ 17.014° N 54.1013° E	Urtica *Urticaceae*	Weed	ChiLCV	Indian subcontinent	ChiLCV	OM326820	OM368457	NAFME5
008-4	2016	Al Suwayq/ 23.8262° N 57.4288° E	Okra *Malvaceae*	Crop	CLCuGeV	Sudan	No begomovirus detected	NA	OM368449	NAFME5
005-2HamFR	2016	Khasab/ 26.1654° N 56.2432° E	Red pumpkin *Cucurbitaceae*	Crop	No begomovirus detected	NA	No begomovirus detected	NA	OM368476	NAFME5
WSM-2CO13FR	2017	Al-Batinah/ 24.2421° N 56.4320° E	Tomato *Solanaceae*	Crop	ToLCBrV	Middle East	ToLCBrV	OM326815	OM368464	NAFME5
010-3	2017	Rustaq/ 23.5058° N 57.2539° E	Eggplant *Solanaceae*	Home garden	No begomovirus detected	NA	No begomovirus detected	NA	844/ OM368455	NAFME5
008-3	2017	Al Buraymi/ 24.1660° N 56.1130° E	Pumpkin *Cucurbitaceae*	Crop	WmCSV	Northern Middle East	No begomovirus detected	NA	OM368453	NAFME5
WSO-1CO13FR	2017	Sohar/ 24.196° N 56.408° E	Muskmelon *Cucurbitaceae*	Crop	ChiLCV	Indian subcontinent	No begomovirus detected	NA	OM368474	NAFME2
WS-2CO13FR	2017	Nizwa/ 22.9371° N 57.5383° E	Pumpkin *Cucurbitaceae*	Crop	TYLCV-OM	Mediterranean and/or Middle East	TYLCV-OM	OM326810	OM368476	NAFME2
WS-3CO13FR	2017	Al-Batinah/ 23.1664° N 57.5432° E	Cucumber *Cucurbitaceae*	Green house	WmCSV/TYLCV-OM	Mediterranean and/or Middle East	TYLCV-OM	OM326809	OM368470	NAFME2
Ham-2FR	2018	Al-Batinah/ 23.1664° N 57.5432° E	Muskmelon *Cucurbitaceae*	Small-scale	WmCSV	Northern Middle East	No begomovirus detected	NA	OM368477	NAFME5
002-2	2018	Khasab/ 26.1644° N 56.2426° E	Cucumber *Cucurbitaceae*	Green house	MYMIV	Indian subcontinent	No begomovirus detected	NA	OM368459	NAFME5
004-2	2018	Al Barka/ 23.6867° N 57.9058° E	Tomato *Solanaceae*	Crop	ChiLCV	Indian subcontinent	ChiLCV	OM326821	OM368466	NAFME5
WK-2CO13FR	2018	Rustaq/ 23.5058° N 57.2519° E	Papaya *Caricaceae*	Garden	TYLCV-OM	Mediterranean and/or Middle East	TYLCV-OM	OM326808	OM368456	NAFME5
WSM-1CO13FR	2018	Samail/ 23.2969° N 57.9731° E	Papaya *Caricaceae*	Garden	ChiLCV	Indian subcontinent	ChiLCV	OM326822	OM368462	NAFME5
WSE-1CO13FR	2018	Al-Batinah/ 24.2420° N 56.4399° E	Tomato *Solanaceae*	Crop	ToLCSDV	North Africa	No begomovirus detected	NA	OM368477	NAFME2
WSL-2CO13FR	2018	Al-Batinah/ 24.2420° N 56.4299° E	Pumpkin *Cucurbitaceae*	Small-scale	ChiLCV	Indian subcontinent	No begomovirus detected	NA	OM368472	NAFME3
WKM-2CO13FR	2018	Al Masnaah/ 23.7474° N 57.6326° E	Watermelon *Cucurbitaceae*	Crop	WmCSV	Northern Middle East	No begomovirus detected	NA	OM368458	NAFME5
WSG-1CO13FR	2018	Nizwa/ 22.9392° N 57.5378° E	Pepper *Solanaceae*	Crop	ChiLCV	Indian subcontinent	ChiLCV	OM326814	OM368451	NAFME5

* Chili leaf curl virus (ChiLCV), cotton leaf curl Gezira virus (CLCuGeV), mungbean yellow mosaic India virus (MYMIV), squash leaf curl virus (SLCuV), tomato leaf curl Barka virus (ToLCBrV), tomato leaf curl Liwa virus (ToLCLwV), tomato leaf curl Sudan virus (ToLCSDV), tomato yellow leaf curl virus-Oman (TYLCV-OM), and watermelon chlorotic stunt virus (WmCSV). ** NA = not detected.

## Data Availability

The data generated during this research have been included in this published article.

## Supplementary Materials

The following supporting information can be downloaded at: https://www.mdpi.com/article/10.3390/insects14030268/s1, Figure S1: SNP profiles; Figure S2: Begomoviruses_sequencesRaXMLtree.
