# Supplementary figures and images for: Native and Non-Native Bemisia tabaci NAFME Haplotypes Can Be Implicated in Dispersal of Endemic and Introduced Begomoviruses in Oman
Source: Insects. 2023 Mar 8;14(3):268. doi: 10.3390/insects14030268 (PMC10056824; doi:10.3390/insects14030268)

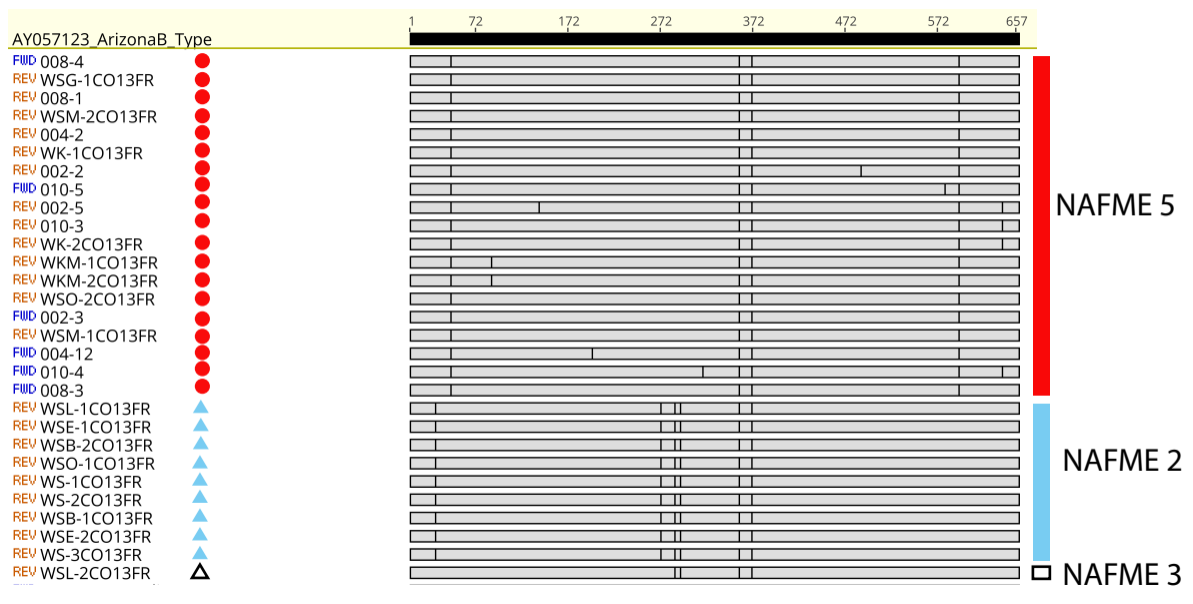

Supplement: Supplementary file 1 [file insects-14-00268-s001.zip › Supplementary Figure S1. SNP profiles.pdf]

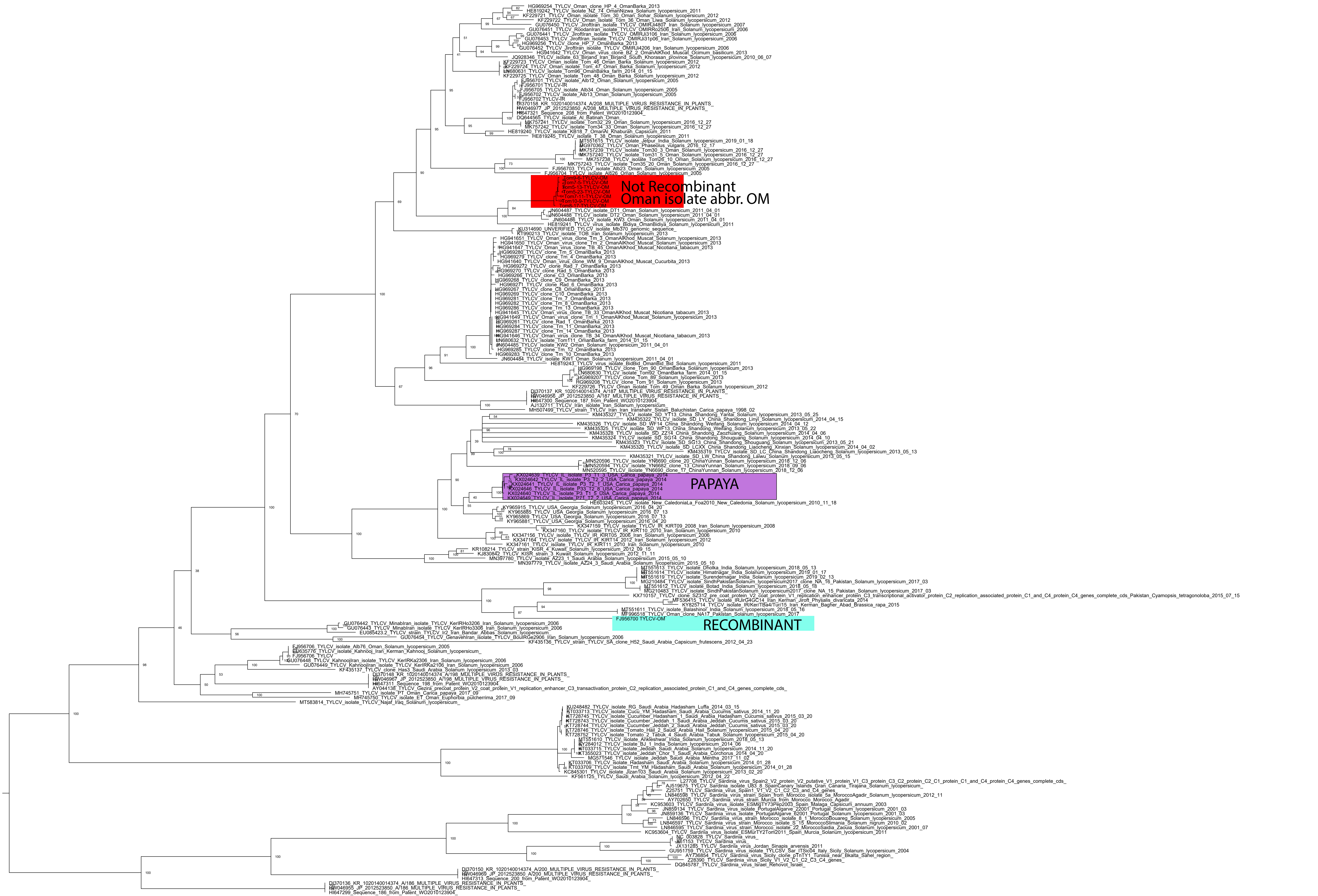

Supplement: Supplementary file 1 [file insects-14-00268-s001.zip › Supplementary Figure S2. TYLCV_sequencesRaXMLtree.pdf]
